# Fe_3_O_4_-Fused Magnetic Air Stone Prepared From Wasted Iron Slag Enhances Denitrification in a Biofilm Reactor by Increasing Electron Transfer Flow

**DOI:** 10.3389/fchem.2022.948453

**Published:** 2022-07-08

**Authors:** Yifeng Wu, Xiangru Liu, Qi Wang, Dongxu Han, Shanshan Lin

**Affiliations:** School of Environment Northeast Normal University, Changchun, China

**Keywords:** iron slag recycling, denitrification, electron transfer enhancer, Fe_3_O_4_-fused air stone, nano Fe_3_O_4_

## Abstract

nFe_3_O_4_ was prepared from waste iron slag and loaded onto air stone (named magnetic air stone or MAS in the following text). The main component of air stone is carborundum. To study the magnetic effects of MAS on denitrification, a biofilm reactor was built, and its microbial community structure and electron transfer in denitrification were analyzed. The results showed that MAS improved the performance of the reactor in both carbon and nitrogen removal compared with air stone (AS) control, and the average removal efficiencies of COD, TN, and NH_4_^+^-N increased by 17.15, 16.1, and 11.58%, respectively. High-throughput sequencing revealed that magnetism of MAS had a significant effect on the diversity and richness of microorganisms in the biofilm. The MAS also reduced the inhibition of rotenone, mipalene dihydrochloride (QDH), and sodium azide on the respiratory chain in denitrification and enhanced the accumulation of nitrite, in order to provide sufficient substrate for the following denitrification process. Therefore, the denitrification process is accelerated by the MAS. The results allowed us to deduce the acceleration sites of MAS in the denitrification electron transport chain.

The existence of MAS provides a new rapid method for the denitrifying electron transport process. Even in the presence of respiratory inhibitors of denitrifying enzymes, the electron transfer acceleration provided by MAS still exists objectively. This is the mechanism through which MAS can restore the denitrification process inhibited by respiratory inhibitors to a certain extent.

## Introduction

The safe and efficient removal of total nitrogen in wastewater has attracted broad attention because of its potential eutrophication effects (Zeng et al., 2018). Compared with a variety of physical and chemical processes for nitrogen removal, the biological process has economic and environmental advantages and is regarded as a better choice (Wang et al., 2009). However, the traditional biological nitrogen removal process is held up by denitrification, which directly affects the efficiency of wastewater treatment facilities. In order to improve denitrification in traditional biological processes, many technologies have been developed, such as anaerobic ammonia oxidation (ANAMMOX) (Sun et al., 2021), short-cut nitrification and denitrification (SND) (Cui et al., 2021; Feng et al., 2021; Li et al., 2021), and oxygen-limited autotrophic nitrification and denitrification (OLAND) (Gao et al., 2017; Nguyen et al., 2017). However, these processes are only used for the side stream sludge treatment but are difficult to be applied to the commercial wastewater treatment facilities because it is challenging to control their intricate running conditions (Ji et al., 2019). Recently, more research has focused on the mechanism of electron transfer to improve the efficiencies of denitrification processes (Guo et al., 2022). Many studies have shown that a magnetic field is beneficial to enhance the activity of microorganisms and improve the efficiency of carbon and nitrogen removal (Yang et al., 2021), but the mechanisms remain unclear.

In addition, the magnetic field affects the microbial community in wastewater treatment systems by changing the diversity and richness of microorganisms, which enhances bacterial activity in the treatment (Liu et al., 2018; Xin et al., 2021). Wang et al. (2017) investigated the effects of magnetic fields of different intensities (0, 5, 10, 15, 20, and 25 mT) on the activity of anammox bacteria (AOB) through short-term batch experiments, which found that magnetic intensity at 5 mT had a significant effect on AOB, and proved that a weak magnetic field is a simple and convenient method to improve the activity of AOB. Ren et al. (2018)found that the magnetic field generated by magnetic powder (Fe_3_O_4_) significantly increased the growth of microorganisms and degradation effectiveness of nitrogen wastewater. It indicates that the addition of Fe_3_O_4_ has a significant impact on the richness and diversity of microorganisms.

The magnetic field also affects the enzyme activity in microorganisms. Mizuki et al. (2010)immobilized the amylase extracted from *Bacillus licheniformis* on the surface of superparamagnetic particles and studied the effect of a rotating magnetic field on enzyme activity; the results showed that the activity of enzyme molecules increased in the rotating magnetic field and reached the maximum value at a certain frequency. Robatjazi et al. (2012)found that the pH stability and intrinsic resistance of the enzyme activity of the magnetically modified bacteria were improved in acidic and alkaline conditions, and the thermal stability of the enzyme could be improved by immobilizing the magnetically modified bacteria with an external magnetic field. The removal of organic matter, nitrogen, and phosphorus is directly related to enzyme activities of microbes in activated sludge and can be significantly affected by the magnetic field (Ma et al., 2017).

It has been demonstrated that the increase in the removal efficiency of total nitrogen with a magnetic field is partly caused by improvement of the microbial community structure and enzyme activities (Ni et al., 2013). However, few studies were carried out on the effects of the magnetic field on electron transport in the denitrification process. The denitrification reductase plays an important role in electron transfer from reduced carbon sources to NADH (or FADH_2_) and finally to the N-containing electron acceptors in the process of denitrification.

Specific denitrifying electron transport chains can be inhibited or blocked by adding different kinds of respiratory inhibitors with a certain dose limit. For example, rotenone is a respiratory chain complex I (NADH dehydrogenase) inhibitor, which can inhibit the NADH to coenzyme Q reductase activity by substituting coenzyme Q intermediates (Xi et al., 2013). QDH is a quinone analog inhibitor that can hinder the transfer of electrons from FADH_2_ to coenzyme Q (Zeng et al., 2018). Sodium azide is an inhibitor of respiratory chain complex IV (cytochrome oxidase). According to reports, sodium azide can also inhibit membrane-bound nitrate reductase (Nar enzyme) (Kraft et al., 2011), which is sensitive to sodium azide in the concentration range of 1 μmol/L (Marangon et al., 2012).

In this study, we demonstrated that adding the Fe_3_O_4_-fused air stone greatly increased denitrification in a biofilm reactor. Further high-throughput sequencing demonstrated that the structure of the microbial community was changed in the presence of magnetism. To further identify the role of nFe_3_O_4_ in denitrification, under the premise that the electron transport chain of denitrification is inhibited, Fe_3_O_4_-fused air stone considerably alleviated the inhibition of electron transfer in respiration, indicating it enhanced electron flow in denitrification, which is significant in identifying and improving the rate-limiting step in nitrogen removal in wastewater treatment.

## Materials and Methods

### Seed Sludge and Synthetic Wastewater

The seed sludge was taken from the secondary sedimentation tank of the sewage treatment plant in Changchun, China. Synthetic wastewater is simulated municipal sewage; the sewage is mainly composed of 308 mg/L NH_4_Cl, 513 mg/L CH_3_COONa, 66 mg/L KH_2_PO_4_, 50 mg/L MgSO_4_, 8 mg/L CaCl_2_·H_2_O, 21.6 mg/L beef extract, 22.8 mg/L peptone, 90.7 mg/L NaHCO_3_ (mainly used to adjust pH), and other necessary trace elements. The trace elements include the following (mg/L): 0.024 NiCl_2_ 6H_2_O, 0.061 MnSO_4_·H_2_O, 0.024 Na_2_MoO_4_ 2H_2_O, 0.1 CoCl_2_ 6H_2_O, 0.07 ZnCl_2_, 0.002 CuCl_2_ 2H_2_O, and 0.006 H_3_BO_3_. During this period, COD remained at about 300 mg/L.

### Preparation of Magnetic Air Stone and Reactor Operating Conditions

FeCl_3_ 6H_2_O and FeCl_2_ 4H_2_O were prepared from waste iron slag by high-temperature roasting and 30% hydrochloric acid leaching, followed by evaporation crystallization. Using the mixture of FeCl_2_ 4H_2_O and FeCl_3_ 6H_2_O recovered from iron slag as raw materials, nFe_3_O_4_ was prepared by the alkaline co-precipitation method and then modified with oleic acid. The modified nFe_3_O_4_ and air stone (AS) were placed in a beaker containing 200 ml n-hexane. We used a vacuum drying oven to immerse at 40°C under negative pressure for half an hour, then sintered in a nitrogen muffle furnace at 200°C and 450°C for 1 h respectively, and finally made into magnetic air stone (MAS) (Liu et al., 2015).

SiC air stone has a Mohs hardness of 9.5, strong oxidation resistance, and does not participate in the denitrification reaction. After detection, the specific surface area of AS is 25.5 and porosity is 82.3%, while that of MAS is 23.8 and porosity is 79.6%. The specific surface area and porosity of MAS are lower than those of AS due to the loading of nFe_3_O_4_.

**FIGURE 1:**
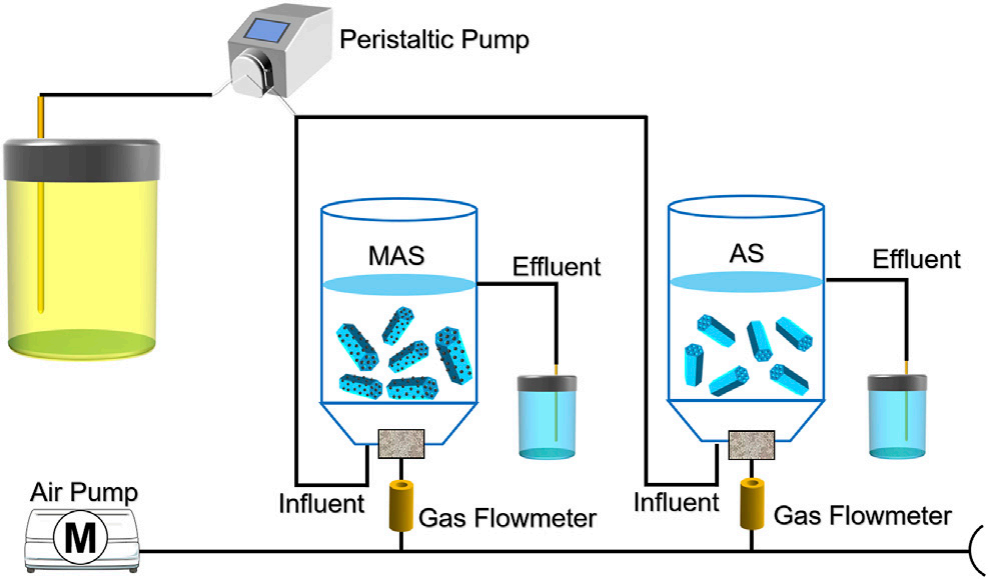
Diagram of the experimental device.

The reaction device is shown in Figure 1 It consists of two plexiglass containers with a diameter of 100 mm, a height of 190 mm, and an effective volume of 1.5 L, a peristaltic pump, aerator head, and gas flow meter. After the activated sludge was domesticated, eight pieces of MAS and AS were added to the reactor, respectively. The aeration head and the water inlet were in concurrent contact at the bottom of the reactor, the air–water ratio is 8:1, and the hydraulic residence time was for 5 h, the temperature was controlled at about 25°C, and the dissolved oxygen was 2∼4 mg L^−1^; then the biofilm was loaded (Liu et al., 2015). After the biofilm matured, water was continuously fed in and out, and the removal efficiencies of COD, TN, and NH_4_^+^-N were monitored every day. The experiment lasted for 110 days.

### DNA Extraction, PCR Amplification, Sequencing, and Data Analysis

Here, 5 g of uniformly mixed biofilm samples were collected and stored at −80°C until DNA extraction (Miao et al., 2017). To ensure the rigor of the experiment, each group of samples was made in triplicate. The DNA was amplified in the V4 region of the 16S rRNA gene, and the gene sequence obtained was analyzed by the Illumina Hiseq 2500 platform. After the quality test, the clean label was obtained. Using USEARCH (V8.0.1517) software, the original label was clustered into different operational classification units, according to 97% of the identity (Zeng et al., 2018). The microbial community structure can be further annotated by matching with Greengenes, SILVA, and UNITE databases. The technology was completed by Biomark Biotechnology Co., Ltd. (Beijing, China).

### Respiratory Inhibitor Dosing Experiment

The experiment was carried out at 25°C to study the effects of different electron acceptors on the denitrification performance. The reaction device with a volume of 250 ml was sealed with a rubber stopper. One port on the stopper took liquid, and the other port used an aeration pump to control the dissolved oxygen concentration at 2∼4 mg/L and injected 100 ml of synthetic wastewater. The electron acceptors were NO_3_^−^-N and NO_2_^−^-N with a concentration of 70 mg/L. The synthetic wastewater used CH_3_COONa as a carbon source, and the C/N ratio and other nutrients were the same as in the 2.1 parent reactor earlier. Samples were taken at 0, 5, 10, 20, and 25 h to measure the COD concentration changes, and each group of experiments was repeated three times.

In order to further analyze the influence of magnetism on the electron transport chain of denitrification, 70 mg/L of NO_3_^−^-N was used as the electron acceptor, CH_3_COONa as the carbon source, and rotenone, QDH, and sodium azide as respiratory chain inhibitors. Rotenone can inhibit complex I (Yu et al., 2015), and the applied concentrations were 0, 0.02, 0.05, and 0.1 mmol/L. QDH with concentrations of 0, 0.1, 0.25, and 0.5 μmol/L was added to inhibit complex II (Yang et al., 2018). Sodium azide was added at concentrations of 0, 0.5, 1, and 5 μmol/L, which could inhibit complex IV (Xi et al., 2013).

### Analytical Methods

COD, TN, NH_4_^+^-N, NO_3_^−^-N, and NO_2_^−^-N were measured according to standard methods (APHA, 1999). The DO and pH were measured with a dissolved oxygen meter (SANXIN, SX700, China). X-ray diffraction (XRD; Rint 2200, Rigaku Corporation, Japan) and scanning electron microscopy (SEM; Sigma 300, Zeiss, Germany) were performed. All water samples were repeated in three groups, and the average value was calculated with the standard deviation.

## Results and Discussion

### Characteristics of Air Stone and Magnetic Air Stone

**FIGURE 2:**
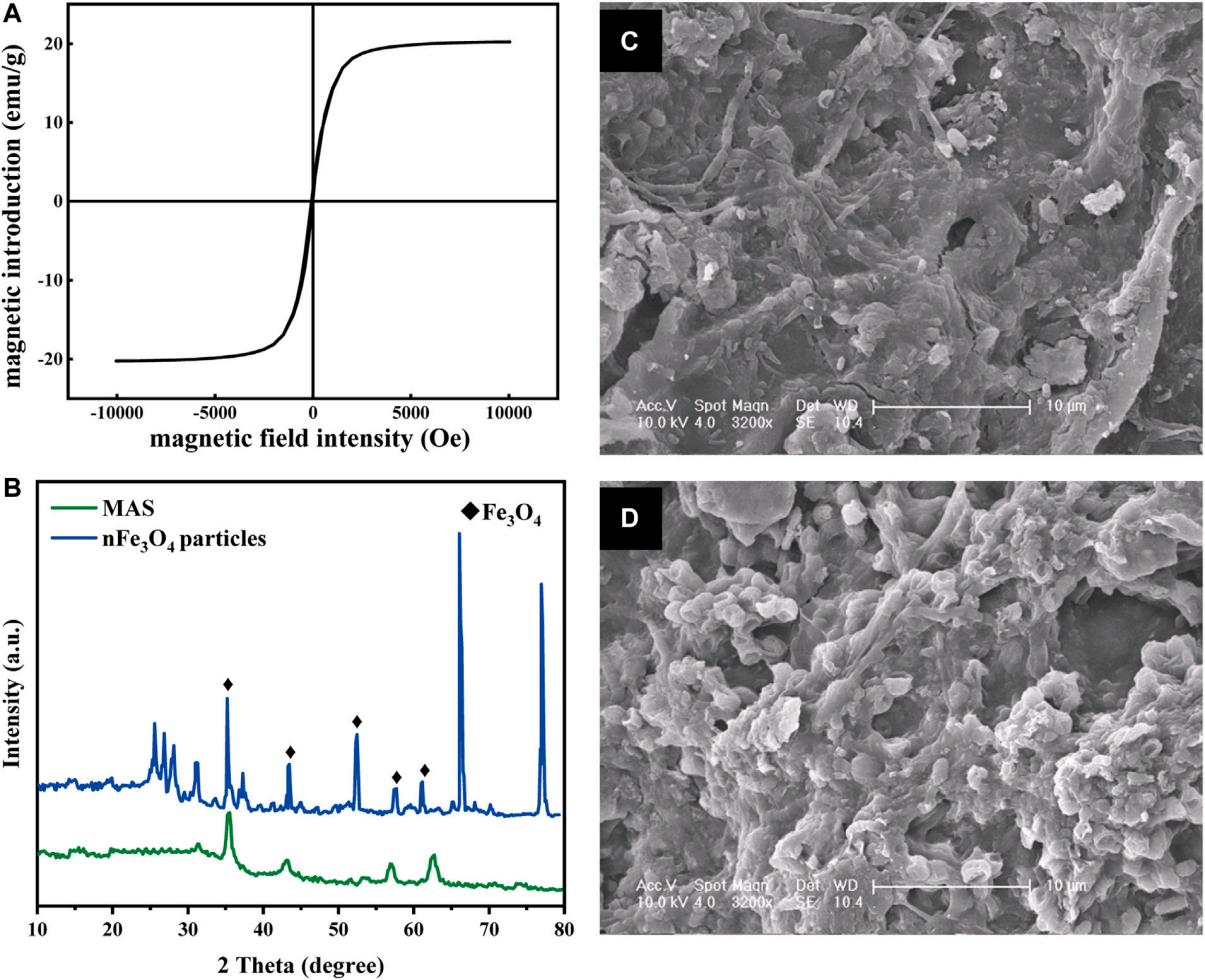
Characterization of MAS. **(A)** Magnetic hysteresis loop of MAS; **(B)** XRD pattern of nFe_3_O_4_ particles (blue) and MAS (green) **(C)** and **(D)** SEM images of biofilms on MAS and AS, respectively (×3200 times).

Figure 2B shows the XRD patterns of nFe_3_O_4_ particles and MAS. Five characteristic peaks appear in the diffraction peaks of these two samples, which are, respectively, at 2θ = 35.5°, 43.3°,53.4°, 57.2°, and 62.5°. After comparison with the standard card, they can be identified as the characteristic peaks of Fe_3_O_4_. This proves that the modified MAS successfully loads nFe_3_O_4_ particles.

The magnetic induction strength of the prepared MAS was measured by using a vibrating sample magnetometer (VSM), and the results are shown in Figure 2A. It was demonstrated that the saturation magnetic induction intensity of MAS had 20 emU/g of superparamagnetic particles (Chen and Wang, 2011), which indicated it had good dispersibility after removing the external magnetic field. Superparamagnetic nFe_3_O_4_ particles are smaller than the critical size (Li et al., 2017); these characteristics make it more fully exposed to pollutants. It has also been reported that such metal oxide nanoparticles have cell membrane permeability (Ajayi-Banji and Rahman, 2021) and microbial affinity (You et al., 2018).

The microstructure of the biofilm on MAS and AS is shown in Figures 2C,D. The shapes of microorganisms growing on the biofilm on MAS and AS were different. The bacteria attached to MAS were mostly rod-shaped, and those attached to AS were almost spherical-shaped. In addition, the rod bacteria on MAS were arranged more compact and orderly than the spherical ones on AS, indicating magnetism might stimulate the growth of different species of microorganisms.

### Influence of Magnetic Air Stone on the Performance of Biofilm Systems

**FIGURE 3:**
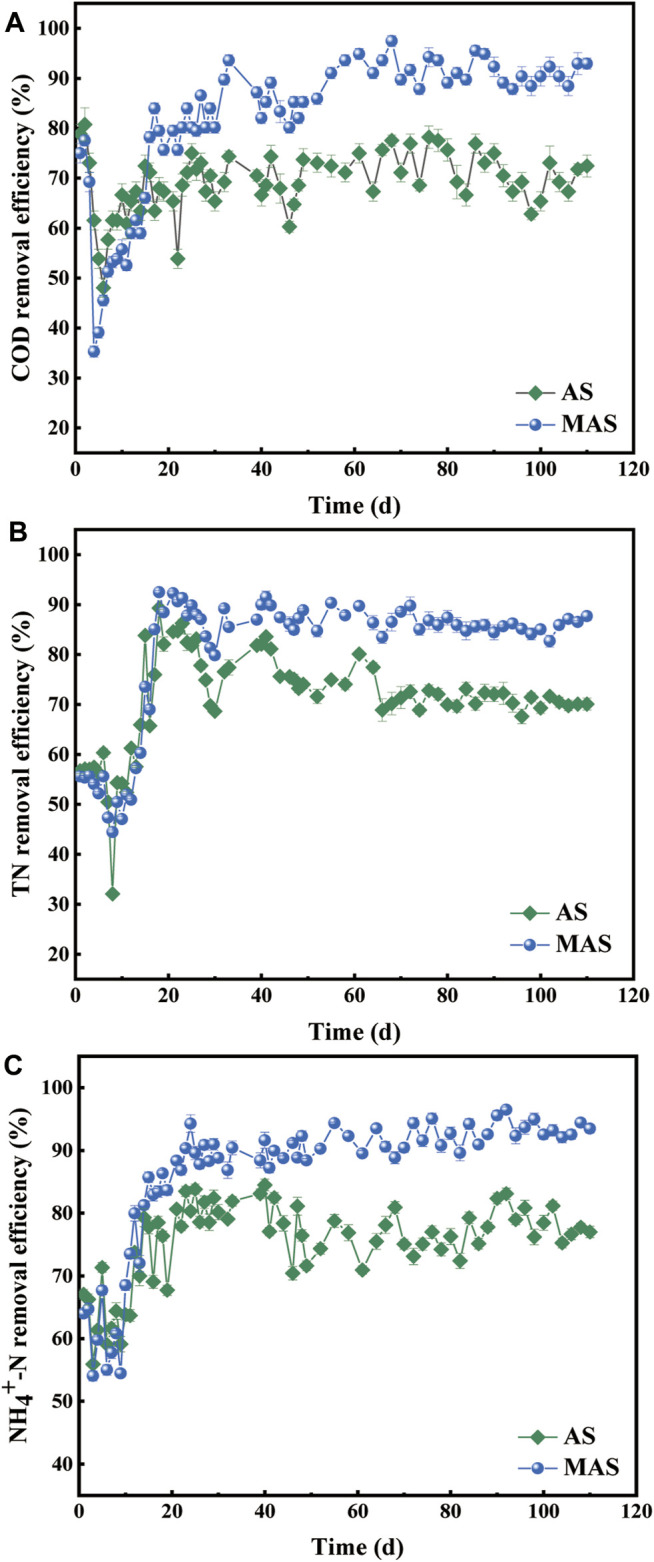
Removal efficiencies of **(A)** COD, **(B)** TN, and **(C)** NH_4_^+^-N with MAS and AS reactors.

The MAS and AS reactors were operated continuously for 110 days. The removal efficiencies of COD, TN, and NH_4_^+^-N were evaluated in the two reactors, respectively (Figure 3). In the first 15 days after inoculation, the removal efficiencies of COD, TN, and NH_4_^+^-N in the two reactors showed similar fluctuating patterns, indicating both reactors were unstable at the initial adapting stage. The removal efficiencies by the MAS reactor were slightly lower because the porosity and specific surface area of MAS were reduced after nFe_3_O_4_ was loaded (Liu et al., 2015). After the initial 15 days, the removal efficiencies of COD, TN, and NH_4_^+^-N in the two reactors became relatively stable. Compared with AS, the average removal efficiency of COD by the MAS reactor increased from 70.21% on which day to 87.36% on which day and reached the highest 97.44% on which day. In the same period, TN and NH_4_^+^-N increased from 70.84% to 86.94% and from 76.25% to 87.83%, respectively, after the reactor was stabilized. During the stable stage (from day 20 to day 110), the MAS increased the average removal efficiency of COD, TN, and NH_4_^+^-N by 17.15, 16.1, and 11.58%, respectively, compared with the AS reactor.

It showed that the MAS promoted the removals of COD, TN, and NH_4_^+^-N after stabilization of the reactor, indicating MAS played a positive role in the removal of carbon and in the nitrification and denitrification processes. nFe_3_O_4_ nanoparticles have inherent magnetic properties, which stimulated the growth and activity of microorganisms by influencing cell energy metabolism and cytoplasmic synthesis (Nakamura et al., 1997). The results were consistent with some other studies, in which magnetic biological effects affected the performance of wastewater biological treatment systems (Lebkowska et al., 2011; Yao et al., 2013). It is also reported that the magnetic field affects microbial growth and substrate degradation in batch reactors (Ma et al., 2017).

### Influence of Magnetic Air Stone on the Microbial Community Structure of the Biofilm System

**FIGURE 4:**
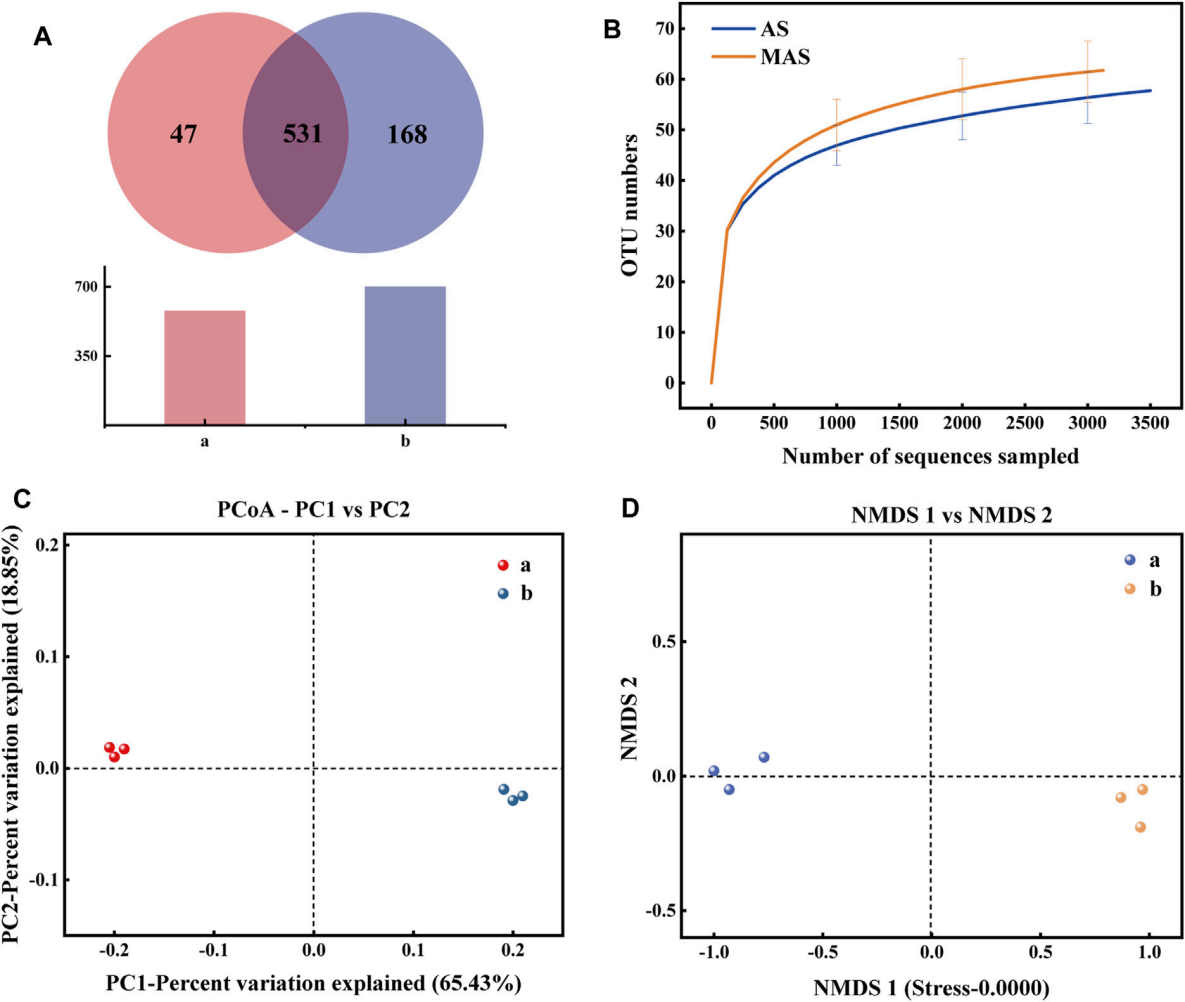
**(A)** Bacteria Venn diagram of two biofilm samples: **(A)**: AS and **(B)**: MAS. **(B)** Dilution curves of two biofilm samples. **(C)** PCoA diagram of two samples: **(A)**: AS and **(B)**: MAS. **(D)** NMDS diagram of two samples: **(A)**: AS and **(B)**: MAS.

**FIGURE 5:**
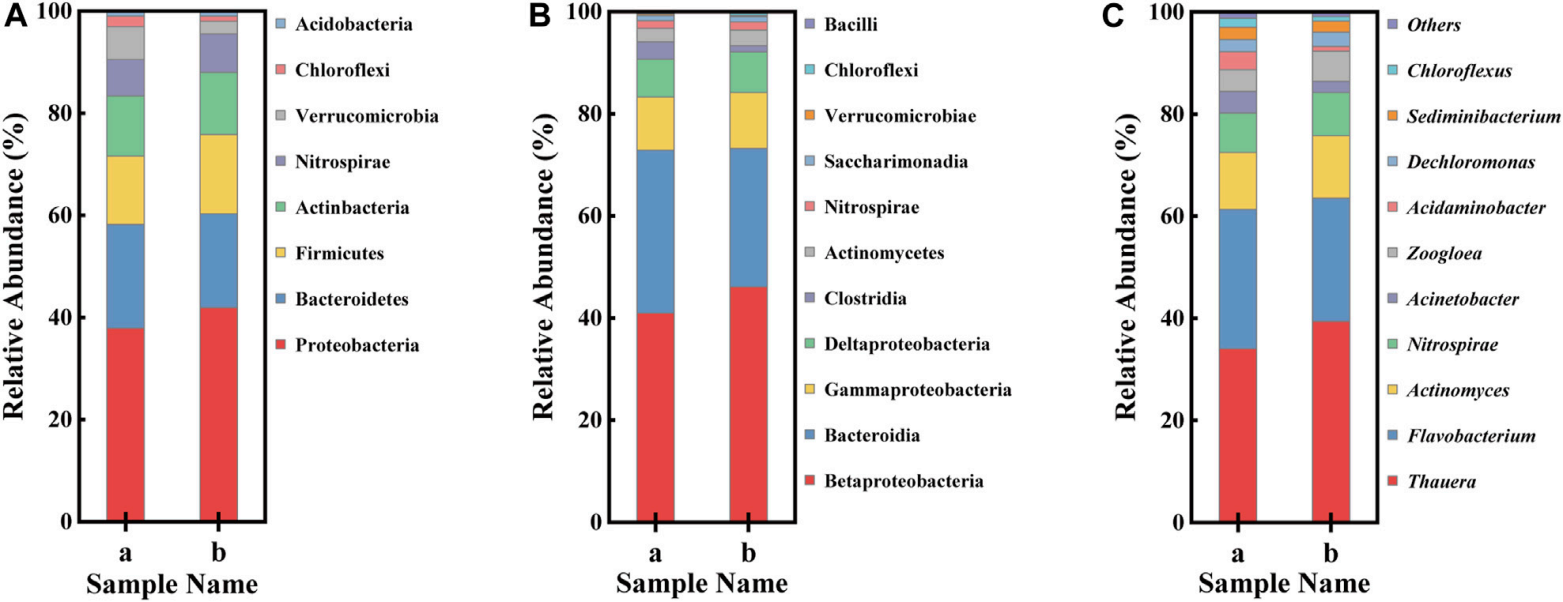
Bar graph of bacterial populations of two samples [**(A)** AS and **(B)** MAS] of the biofilm [**(A)** Phylum, **(B)** class, and **(C)** genus].

In order to better understand the influence of magnetism on microbial richness and diversity, high-throughput sequencing was used to measure the species richness of biofilms grown on AS and MAS in Figure 4. The microbial communities in the biofilms of the two reactors were analyzed at the levels of phylum, class, and genus, and the result is shown in Figure 5.

According to Figure 4A, there are 531 OTUs in the two biofilm samples, accounting for 91.87% and 75.97% of the total OTUs in each sample, respectively, which indicates that the types of these bacteria are not affected by magnetism. The numbers of OTUs unique to ordinary and MAS biofilms were 47 and 168, respectively, accounting for 8.13% and 24.03% of the total OTU, indicating that more types of bacteria were generated after the introduction of magnetism, that is, magnetism increased the diversity of bacteria. The electron microscope shows that under a static magnetic field of 20 mT, the denitrifying sludge will change the diversity of its composition and cell morphology (Benyoucef et al., 2021). According to another report, the static magnetic field is considered as a factor that affects DNA integrity, mutation, transcription, and translation (Amara et al., 2007). All these prove that a new species can be induced by magnetic force.

Table 1 shows the diversity estimation of these two samples by Chao 1, Ace, Shannon, Simpson, and Good’s coverage. Among them, the Chao 1 index and Ace index reflect the richness of the community. The higher the value, the higher is the richness of the community. The Shannon index and Simpson index reflect the diversity of the community, and the larger their value is, the higher the diversity of the samples is. In this study, Good’s coverage rate of all samples was higher than 0.997, proving that the sequencing depth can indicate the true microbial community of the two samples. From Table 1, it can be seen that all indexes of magnetic foam stone are larger than those of ordinary foam stone, indicating that the richness and diversity of bacteria in the magnetic foam stone biofilm are higher. Some studies show that magnetic fields can affect the stability of DNA and directly interact with DNA (Li and Chow, 2001). Dilution curves can also be used to verify whether the amount of sequencing data is enough to reflect the species diversity and indirectly reflect the species richness in the samples. The sequencing depth in Figure 4B can reflect the diversity of species in samples AS and MAS. Analysis of β diversity in Figures 4C,D compared the degree of similarity in the species diversity of AS and MAS. In PCoA analysis and NMDS analysis, it was found that the two samples are apart, which indicates that the microbial structures of AS and MAS are different to some extent, and this difference has not yet been quantified.

**TABLE 1:** Diversity index of two samples.

	Chao1	Ace	Shannon	Simpson	Coverage
AS	3.72	59.5	5.97	0.85	0.998
MAS	3.81	68.1	6.49	0.86	0.999

It was demonstrated that proteobacteria, betaproteobacteria, and Thauera were the most dominant in the phylum, class, and genus levels in both MAS and AS reactors (Figure 5), suggesting Thauera was the dominant species in both reactors. Thauera largely exists in wet soil and polluted freshwater and is the important bacteria in denitrification (Xu et al., 2018). The results indicated that even though the reactors were aerated, denitrifiers developed well in the anaerobic layer of the biofilm, and denitrification proceeded without interruption, which was consistent with the TN removal results (Figure 3B). The dominant Thauera demonstrated slightly higher in MAS than in AS, indicating the MAS may have played a role in promoting the growth of the organisms. It was reported that the magnetic field intensity of 3.2 mT effectively enhanced the microbial fermentation process (Chen et al., 2021). It can also be speculated that the weak magnetic property of MAS is at work. The second abundant ca categories in the MAS and AS reactors are bacteroidetes, bacteroidia, and *Flavobacterium* at the phylum, class, and genus levels, respectively. Flavobacteria are commonly identified in various soil and freshwater environments. It belongs to the heterotrophic denitrifying bacteria and carries out denitrification under anaerobic or hypoxic conditions. The two categories accounted for over 55% of the total identified organisms, indicating they are the major organisms in denitrification.

At the genus level, relatively abundant bacteria are *Thauera*, *Flavobacterium*, *Actinomyces*, *Nitrospirae*, and *Acinetobacter*. In addition, there are some relatively low-abundance bacteria such as *Zoogloea* and *Sediminibacterium*. After AS was magnetically modified, *Thauera*, *Actinomyces*, *Nitrospirae*, and *Zoogloea* all increased to varying degrees, while the relative abundance of *Flavobacterium*, *Acinetobacter*, and *Sediminibacterium* decreased from 27.25, 4.23, 2.43–24.09, 2.17, and 2.16%. *Thauera* belongs to the *Proteobacteria*, which has the ability to denitrify. *Flavobacterium* is a kind of *Bacteroides*, named for its ability to denitrify under anaerobic or hypoxic conditions, and belongs to heterotrophic denitrifying bacteria (Cynthia, T., 2010). *Actinomyces* is also a heterotrophic anaerobic type, with denitrification that gradually converts nitrate to nitrogen and helps the removal of COD (Shu et al., 2015). *Nitrospirae* belongs to nitrifying bacteria and is closely related to the conversion of nitrite to nitrate (Hu et al., 2020). *Acinetobacter* belongs to phosphorus-accumulating bacteria. According to research, *Zoogloea* is widely present in activated sludge and biofilm and is considered to be a key species for activated sludge flocculation (Zhang et al., 2012). The presence of magnetism increased the relative abundance of *Zoogloea* and the formation of biofilms, which was conducive to the formation and maintenance of biofilms, and played a catalytic role in the nitrogen removal of biofilms (Martin et al., 2016).

### Influence of nFe_3_O_4_ on the Denitrification Performance With NO_3_^−^-N or NO_2_^−^-N as the Electron Acceptor

A lot of practices and research tell us that in the traditional microbial denitrification process, an organic carbon source is the main energy of microbial denitrification (Yang et al., 2020). The denitrification can remove COD in raw water, so the efficiency of denitrification can be indirectly measured by the removal rate of COD.

**FIGURE 6:**
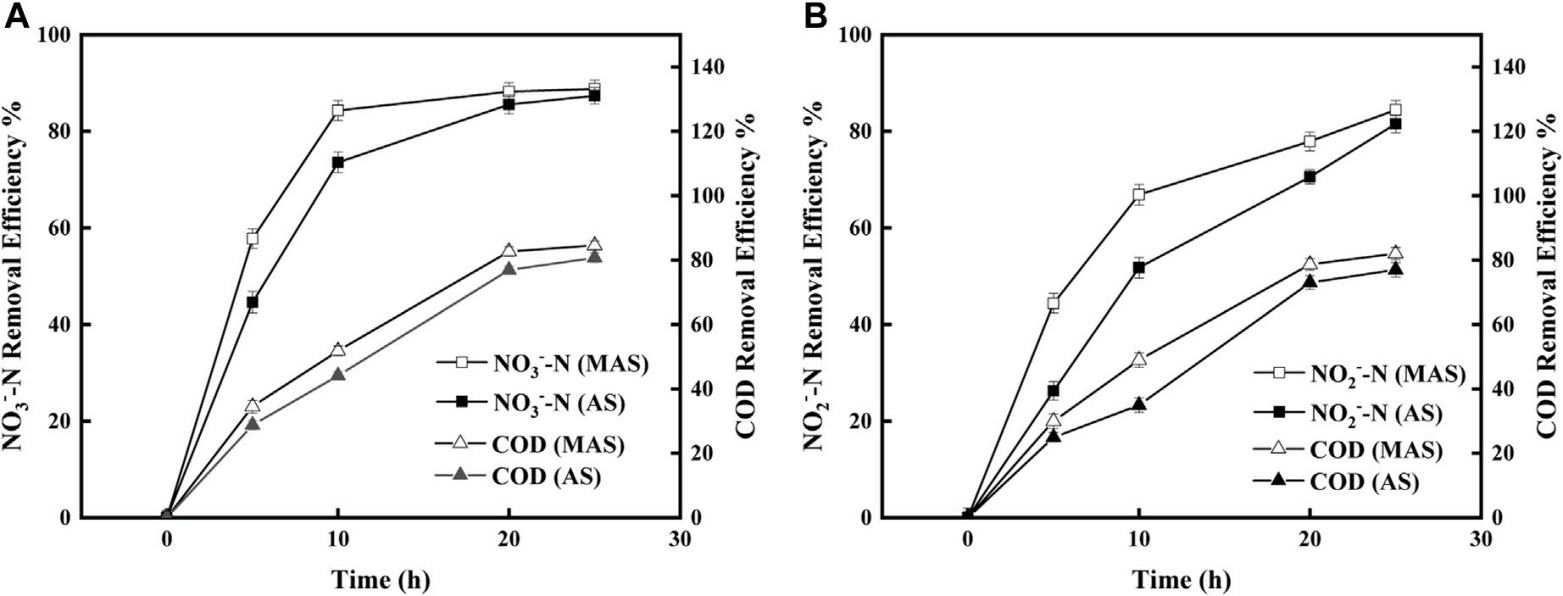
Time course of COD changes in MAS and AS reactors under different denitrification substrates [**(A)** NO_3_^−^-N and **(B)** NO_2_^−^-N].

To identify the function of nFe_3_O_4_ in denitrification, COD degradation was carried out with MAS and AS, respectively, using either NO_3_^−^-N or NO_2_^−^-N as the final electron acceptor. When NO_3_^−^-N was used the AS denitrification substrate, at 10 h, the COD removal efficiency of MAS was 51%, which was higher than that of AS, which was 44%, and the difference was statistically significant after the p-test (*p* < 0.05). Also, at 10 h, when NO_2_^−^-N was used as the denitrification substrate, the COD removal efficiency of MAS was 49%, which is higher than that of AS (35%) (*p* < 0.05). The difference is mainly reflected at 10 h. When the denitrification was nearly finished (at 20 and 25 h), no obvious difference in the COD removal efficiency could be observed in the two substrates. A better COD removal efficiency was observed in MAS with both NO_3_^−^-N and NO_2_^−^-N (Figures 6A,B), indicating MAS facilitates COD degradation by microorganisms.

Meanwhile, denitrification of both NO_3_^−^-N and NO_2_^−^-N was improved with the addition of MAS, indicating reduction of oxidized nitrogen is facilitated by nFe_3_O_4_ (Figures 6A,B). It suggested that nFe_3_O_4_, as an electron carrier, may participate in the transfer of electrons, directly or indirectly, from the substrate to the final electron acceptors. Su et al. (2021)observed the investigation of the mechanism and indicated that the lower electrochemical impedance of Fe_3_O_4_ made it more effective for promoting the intracellular electron transfer of acetoclastic methanogenesis.

### Effect of nFe_3_O_4_ on the Inhibited Respiratory Chain in Denitrification

To further identify the role of nFe_3_O_4_ in denitrification, different inhibitors were used to block different steps in the respiratory chain of denitrification. Rotenone is a type of isoflavones, which is the major component of naturally occurring isoflavonoids (Kaufman et al., 1997). It arrests enzyme complex I (NADH dehydrogenase) in the bacterial respiratory chain, which inhibits the electron transfer step from NADH to coenzyme Q by substituting coenzyme Q intermediates (Xi et al., 2013). QDH is a quinone analog. It inhibits the electron transfer step from FADH_2_ to coenzyme Q (Zeng et al., 2018). Sodium azide is a strong ionic substance. It inhibits enzyme complex IV (cytochrome oxidase) and membrane-bound nitrate reductase (Nar enzyme) (Kraft et al., 2011) at a concentration of 1 μmol/L (Marangon et al., 2012).

**FIGURE 7:**
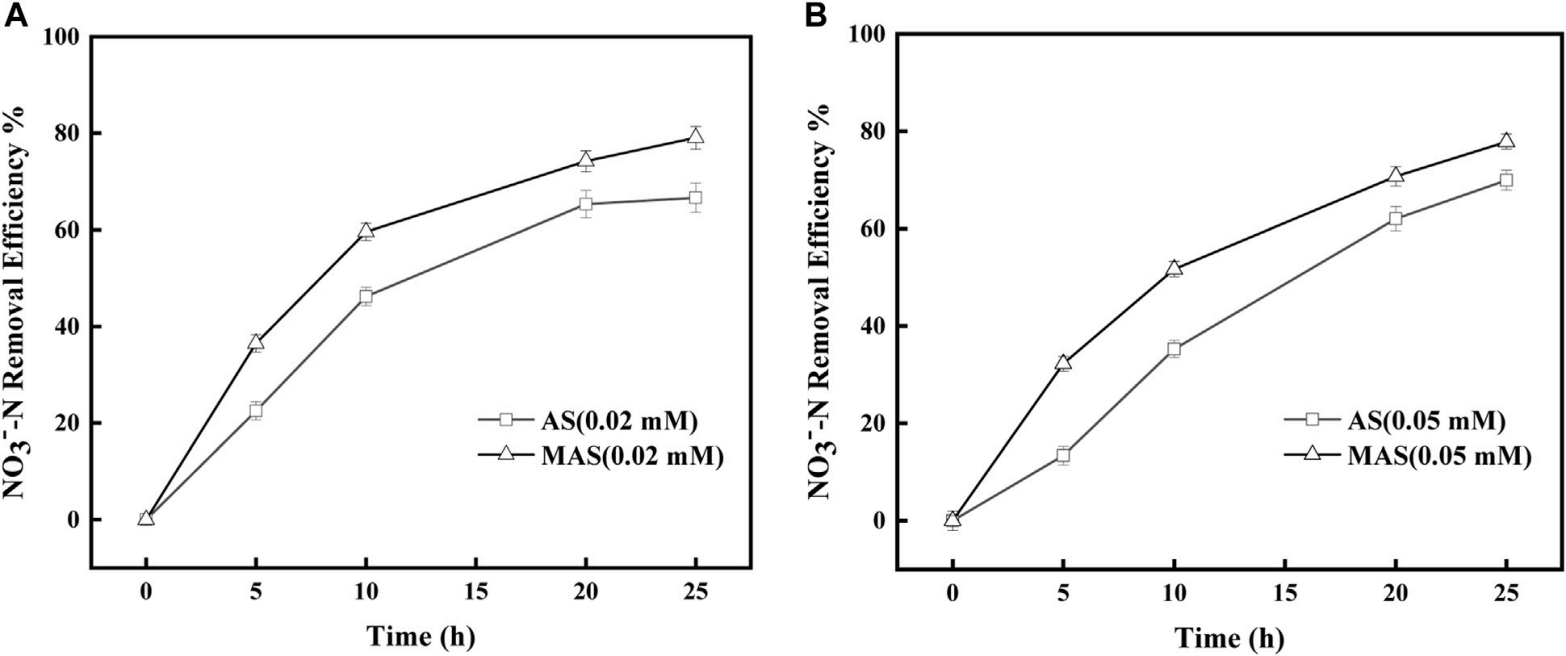
Effect of nFe_3_O_4_ on the rotenone-inhibited respiratory chain in denitrification. Rotenone concentrations were **(A)** 0.02 mM and **(B)** 0.05 mM, and NO_3_^−^-N was used as the final electron acceptor.

With rotenone concentrations of 0.02 and 0.05 mM, the removal efficiencies of NO_3_^−^-N were higher with MAS than those with AS (Figures 7A,B). The NO_3_^−^-N removal efficiencies were 40, 60, 70, and 80%, respectively, with MAS higher than those of 20, 45, 60, and 62% with AS with 0.02 mM of rotenone, on 5, 10, 20, and 25 h (Figure 7A), and the differences were significant with p-test (*p* < 0.05). Similar results were observed with 0.05 mM of rotenone. With the same interval, the NO_3_^−^-N removal efficiencies were 35, 50, 65, and 70, respectively, with MAS higher than those of 15, 30, 55, and 60% (Figure 7B), and the p-test was significant (*p* < 0.05). The results indicated when enzyme complex I of the respiratory chain was suppressed, nFe_3_O_4_ was able to restore the electron transfer in the system, suggesting nFe_3_O_4_ might play a role in the electron transfer chain, which was consistent with the results in Figure 6. Some studies have shown that core-shell nanocomposites are superior for the fabrication of electrochemical sensors (Liu et al., 2013).

**FIGURE 8:**
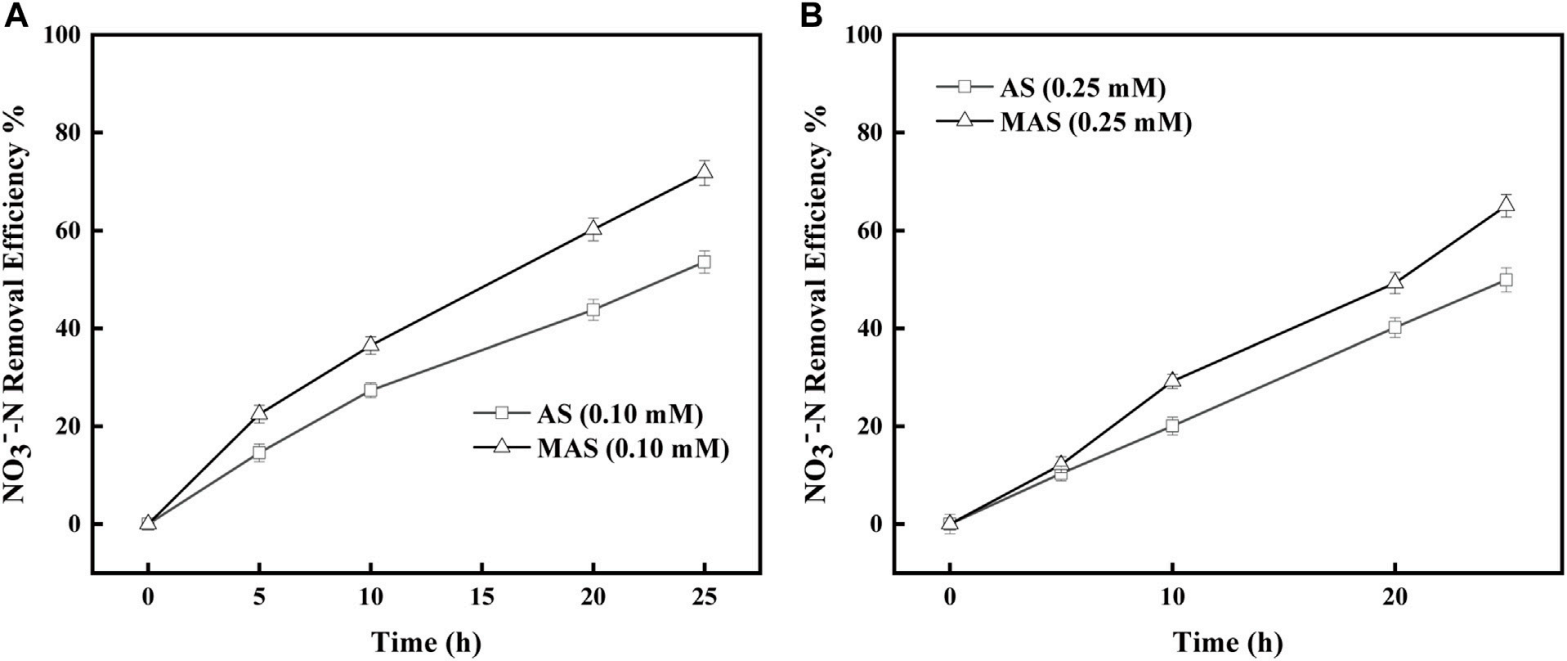
Effect of nFe_3_O_4_ on the QDH-inhibited respiratory chain in denitrification. QDH concentrations were **(A)** 0.1 mM and **(B)** 0.25 mM, and NO_3_^−^-N was used as the final electron acceptor.

Similar inhibitory studies were conducted with QDH, which inhibited enzyme complex II in the respiratory chain and hindered electron transfer from FADH2 to CoQ. The removal efficiencies of NO_3_^−^-N were higher with MAS than those with AS when QDH values were 0.1 and 0.25 mM, respectively (Figures 8A,B). With 0.1 mM of QDH, the NO_3_^−^-N removal efficiencies were X1, X2, X3, and X4%, respectively, with MAS higher than those of Y1, Y2, Y%, and Y4% with AS on 5, 10, 20, and 25 h (Figure 8A), and the differences were significant with the p-test (*p* < 0.05). Similar results were observed with 0.25 mM of QDH. With the same interval, the NO_3_^−^-N removal efficiencies were x1, x2, x3, and x4% with MAS higher than those of y1, y2, y3, and y4% with AS, respectively (Figure 8B), and the p-test was significant (*p* < 0.05). The results indicated when enzyme complex II of the respiratory chain was suppressed, nFe_3_O_4_ was able to restore the electron transfer in the system, suggesting that nFe_3_O_4_ might play a role in the electron transfer chain, which was consistent with the results in Figure 6.

**FIGURE 9:**
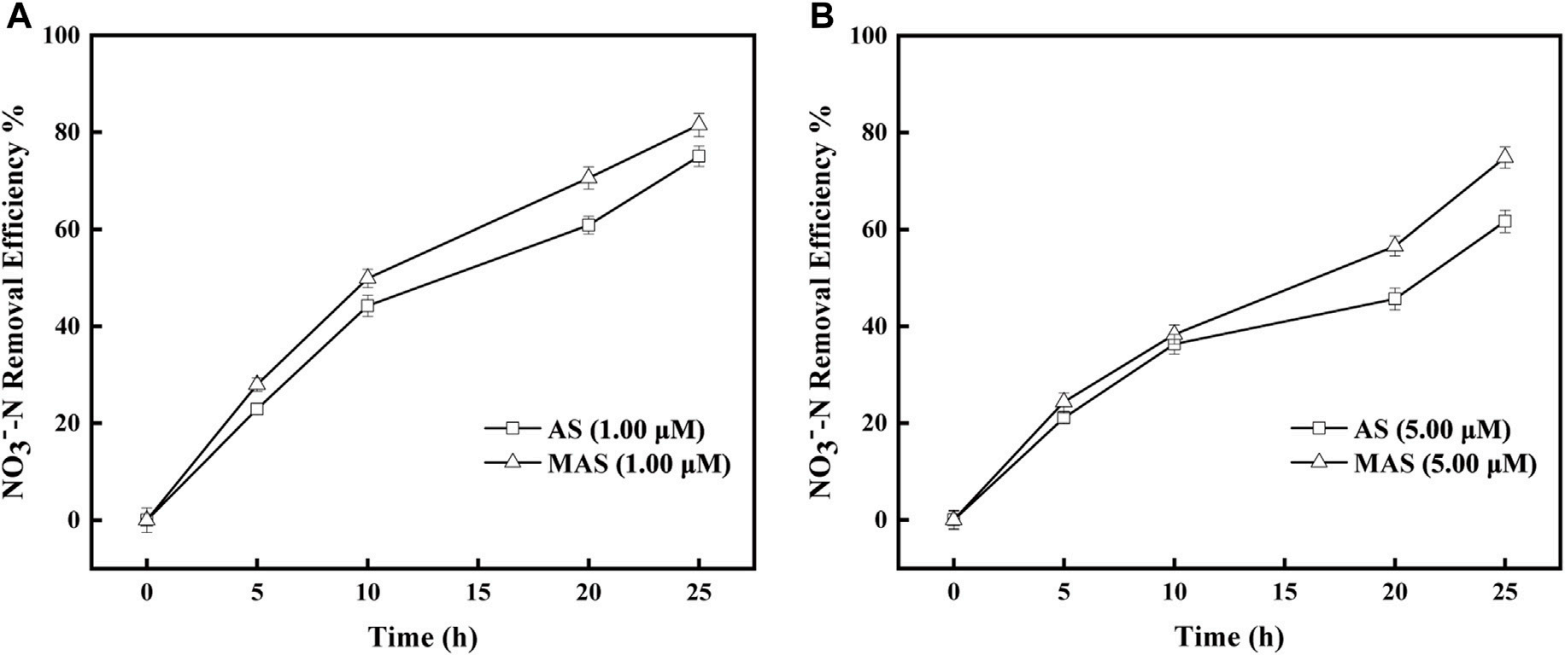
Effect of nFe_3_O_4_ on the sodium azide-inhibited respiratory chain in denitrification. The concentrations of sodium azide were **(A)** 1.00 μM and **(B)** 5.00 μM, and NO_3_^−^-N was used as the final electron acceptor.

The inhibitory studies were also conducted with sodium azide, which inhibited Nar enzyme and interrupted electron transfer from NO_3_^−^-N to NO_2_^−^-N in the respiratory chain. The removal efficiencies of NO_3_^−^-N were higher with MAS than those with AS when sodium azide concentrations were 1 and 5 μM, respectively (Figures 9A,B). With 1 μM of sodium azide, the NO_3_^−^-N removal efficiencies were X1%, X2%, and X3%, respectively, with MAS higher than those of Y1, Y2, and Y3 with AS on 10, 20, and 25 h (Figure 8A), and the differences were significant with the p-test (*p* < 0.05). Similar results were observed with 5 μM of sodium azide; the NO_3_^−^-N removal efficiencies were x1% and x2% with MAS, higher than those of y1% and y2% with AS, respectively on 20 and 25 h, respectively, (Figure 9B), and the p-test was significant (*p* < 0.05). The results indicated when the Nar enzyme of the respiratory chain was suppressed, nFe_3_O_4_ was able to restore the electron transfer in the system, suggesting that nFe_3_O_4_ might play a role in the electron transfer chain, which was consistent with the results in Figure 6.

**FIGURE 10:**
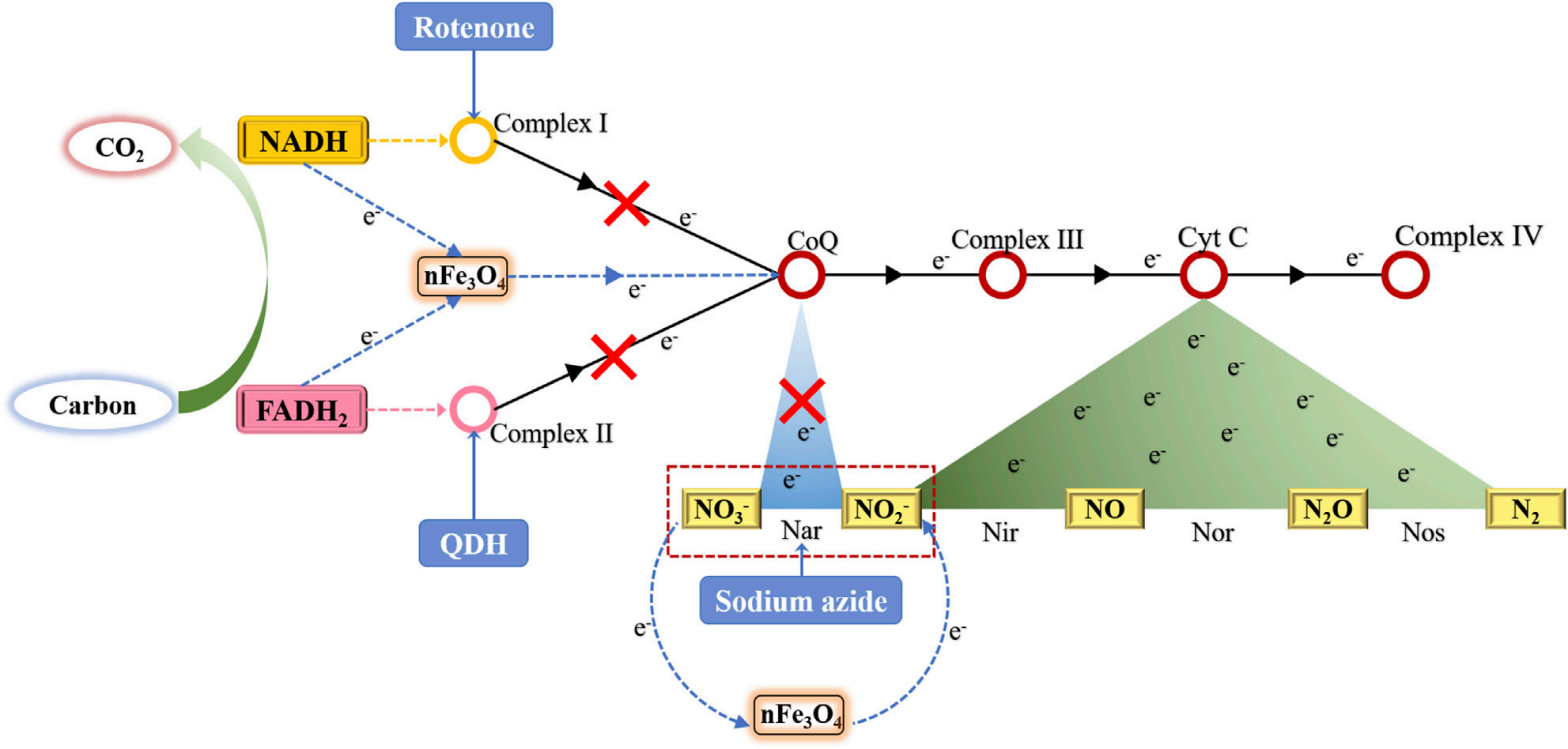
Electron transfer chain in denitrification and the possible role of electron transfer of MAS-nFe_3_O_4_ in the system.

By using three different inhibitors to block three different locations in the microbial respiratory chain, the NO_3_^−^-N removal efficiencies were all improved with MAS, indicating nFe_3_O_4_ played a role in the electron transfer in the respiratory chain in denitrification. It may form an independent electron transfer pathway or facilitate the existing pathways in electron transfer (Figure 10).

## Conclusion

nFe_3_O_4_ was synthesized from scrap iron slag and loaded onto the air stone. The synthesized nFe_3_O_4_-fused MAS improved COD, TN, and NH_4_^+^-N removal efficiencies. It was demonstrated that the richness and diversity of denitrifiers were significantly enhanced in the MAS reactors. Further metabolism and respiratory chain studies with and without inhibitors indicated that nFe_3_O_4_ played a significant role in the first three stages of electron transfer in the microbial respiratory chain (refer to Figure 10, including from NADH to coenzyme from FADH_2_ to coenzyme and from NO_3_^−^-N to NO_2_^−^-N). Future studies will be carried out to prove the existence of a new denitrifying electronic pathway constructed by nFe_3_O_4_ with MAS. In order to achieve this proof, it is hoped that the electronic tracer technology can be used to prove it.

## Data Availability

The datasets presented in this study can be found in online repositories. The names of the repository/repositories and accession number(s) can be found at: NCBI, SAMN28572131.
